# Floral structure and ontogeny of *Syndiclis* (Lauraceae)

**DOI:** 10.1371/journal.pone.0186358

**Published:** 2017-10-13

**Authors:** Gang Zeng, Bing Liu, David K. Ferguson, Jens G. Rohwer, Yong Yang

**Affiliations:** 1 State Key Laboratory of Systematic and Evolutionary Botany, Institute of Botany, the Chinese Academy of Sciences, Beijing, China; 2 University of the Chinese Academy of Sciences, Beijing, China; 3 Department of Paleontology, University of Vienna, Vienna, Austria; 4 Biozentrum Klein Flottbek, Hamburg, Germany; The National Orchid Conservation Center of China; The Orchid Conservation & Research Center of Shenzhen, CHINA

## Abstract

Generic delimitation in the *Beilschmiedia* group of the Lauraceae remains ambiguous because flowering specimens of a few genera with confined distribution are poorly represented in herbaria, and a few floral characters important for taxonomy are still poorly known. *Syndiclis* is sporadically distributed in southwestern China, and is represented in the herbaria by only a few flowering specimens. We conducted field investigations to collect floral materials of four species and observed structures and ontogeny of the tiny flowers using both light microscopy (LM) and scanning electron microscopy (SEM). The results show that the genus *Syndiclis* possesses flowers with huge variation in both merosity and organ number. Flowers of the genus are dimerous, trimerous, or tetramerous, or have mixed merosity with monomerous and dimerous, or dimerous and trimerous, or trimerous and tetramerous whorls. The number of staminodes ranges from two to eight, depending on floral merosity, and on how many stamens of the third androecial whorl are reduced to staminodes. The staminodes of the fourth androecial whorl are comparable to the staminodes in *Potameia*, but the staminodes of the third androecial whorl of *Syndiclis* are relatively larger than the staminodes in *Potameia*. They are erect or curved inwards, covering the ovary. The anthers are usually two-locular, but rarely one-locular or three-locular. Each stamen of the third androecial whorl bears two conspicuous and enlarged glands at the base. The lability of floral merosity and organ number of *Syndiclis* may have been caused by changes of pollination system and loss of special selective pressures that are present in most Lauraceous plants with fixed floral organ number. This study furthers our understanding of variation and evolution of a few important characters of the *Beilschmiedia* group and provides essential data for a revised generic classification of the group.

## Introduction

The Lauraceae have a pantropical distribution in Tropical America, Asia, Australia, and Africa [1,2]. This family consists of ca. 55 genera, and 2500–3500 species with two diversity centres in Tropical America and Tropical Asia [2,3]. Many genera in the family are ill-defined, resulting in many generic complexes or groups, e.g. the *Litsea* group [4], the *Ocotea* group [5], the *Persea* group [6,7], and the *Beilschmiedia* group [8–10].

The *Beilschmiedia* group is monophyletic and sister to *Cryptocarya* R. Br. These taxa form the tribe Cryptocaryeae together with a few other genera including *Eusideroxylon* Teijsm. et Binnend., *Potoxylon* Kosterm., *Aspidostemon* Rohwer et Richter, and *Dahlgrenodendron* van der Merwe et van Wyk [8–10]. The *Beilschmiedia* group was recognized because its taxa share a combination of characters, e.g. the ultimate cymes of inflorescences having alternate/subopposite lateral flowers, the stamens usually possessing two-locular anthers, the tepals usually deciduous and the fruits sitting free on naked pedicles, or rarely the tepals persistent at the base of fruits [11–13]. The *Beilschmiedia* group contains six to nine genera depending on different generic delimitations: *Beilschmiedia* Nees, *Brassiodendron* Allen, *Endiandra* R. Br., *Hexapora* Hook. f., *Potameia* Thouars, *Sinopora* J. Li et al., *Syndiclis* Hook. f., *Triadodaphne* Kosterm., and *Yasunia* van der Werff. Some of the genera were considered synonyms or separated by different authors, but were not yet represented in phylogenetic trees, e.g. *Brassiodendron* and *Hexapora* [2,10,12,13–15]. Taxonomy of the *Beilschmiedia* group remains poorly resolved, especially the generic delimitation.

**Fig 1:**
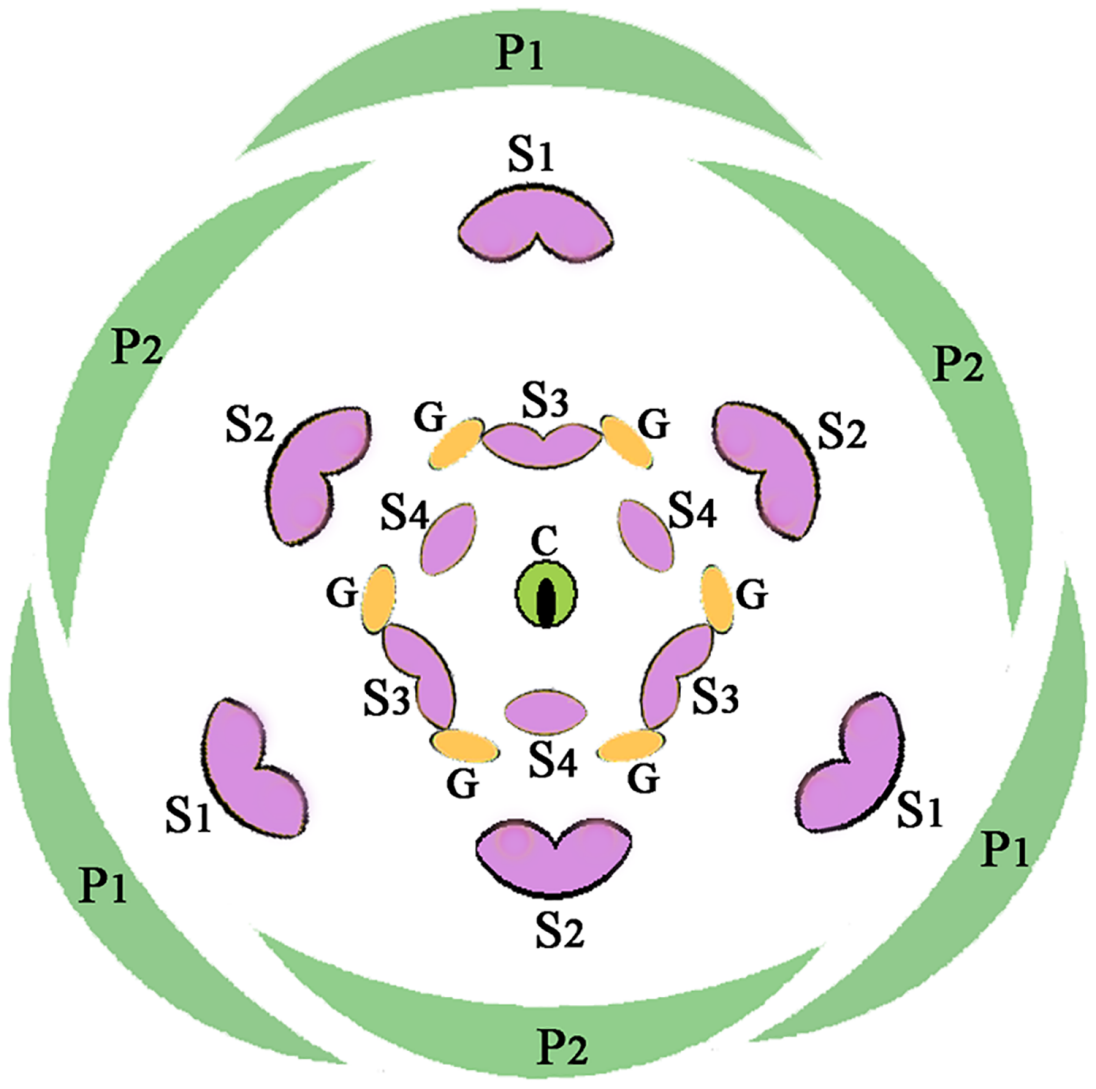
A generalized diagram of typical flowers in the Lauraceae. *Abbreviations*. C, carpel; G, gland; P1, the outer whorl of tepals; P2, the inner whorl of tepals; S1, the first/outermost whorl of stamens; S2, the second whorl of stamens; S3, the third androecial whorl including either fertile stamens or staminodes; S4, the fourth androecial whorl including staminodes.

Typical flowers of the Lauraceae are trimerous, possess two whorls of tepals, three whorls of stamens with the outer two whorls introrse and eglandular, the inner third whorl extrorse and with two glands at the base of each stamen, a fourth whorl of staminodes, and a central pistil (Fig 1). In the *Beilschmiedia* group, the floral structure shows a huge diversity, and floral characters have been used to define the genera in this group, e.g. number of tepals, stamens, staminodes, and anther locules, and position of glands [12,13]. Taxonomic utility of these characters, however, needs to be clarified on account of a few reasons. First, flowering specimens are poorly represented in the herbaria; ca. 1/3 of the Chinese species are imperfectly known due to lacking fruit or flower characters. Therefore, it is necessary to collect floral materials in the field. The genus *Syndiclis* is particularly poorly represented in the herbaria. Second, the flowers of most species in this group are rather small (< 3 mm diam.), so that it is difficult to locate, observe, and dissect these minute flowers. As a result, to better understand the morphological variation of these genera, it is necessary to conduct field investigations and anatomical observations.

Here we report the results of our studies on *Syndiclis* and compare *Syndiclis* with other genera of the *Beilschmiedia* group. *Syndiclis* contains ca. 10 species and is sporadically distributed in Bhutan and southern China [14]. The genus was sometimes considered to be synonymous to a Malagasy genus of the *Beilschmiedia* group, i.e. *Potameia*, due to their similarity in floral morphology [1,2], though they were not so close as expected according a recent molecular phylogeny (unpubl. data). For comparison, we also observed flowers of four species of *Potameia*.

## Materials and methods

The field investigations were conducted in collective forests in Malipo County, Napo County and Maguan County of Yunnan Province, China. These forests are owned by the local villages. We got permission from local villagers (Mr. Xibin Guo, Mr. Yingchao Zhao, Mr. Xinghui Huang, Mr. Mingde Chen) to collect materials and specimens.

**Fig 2:**
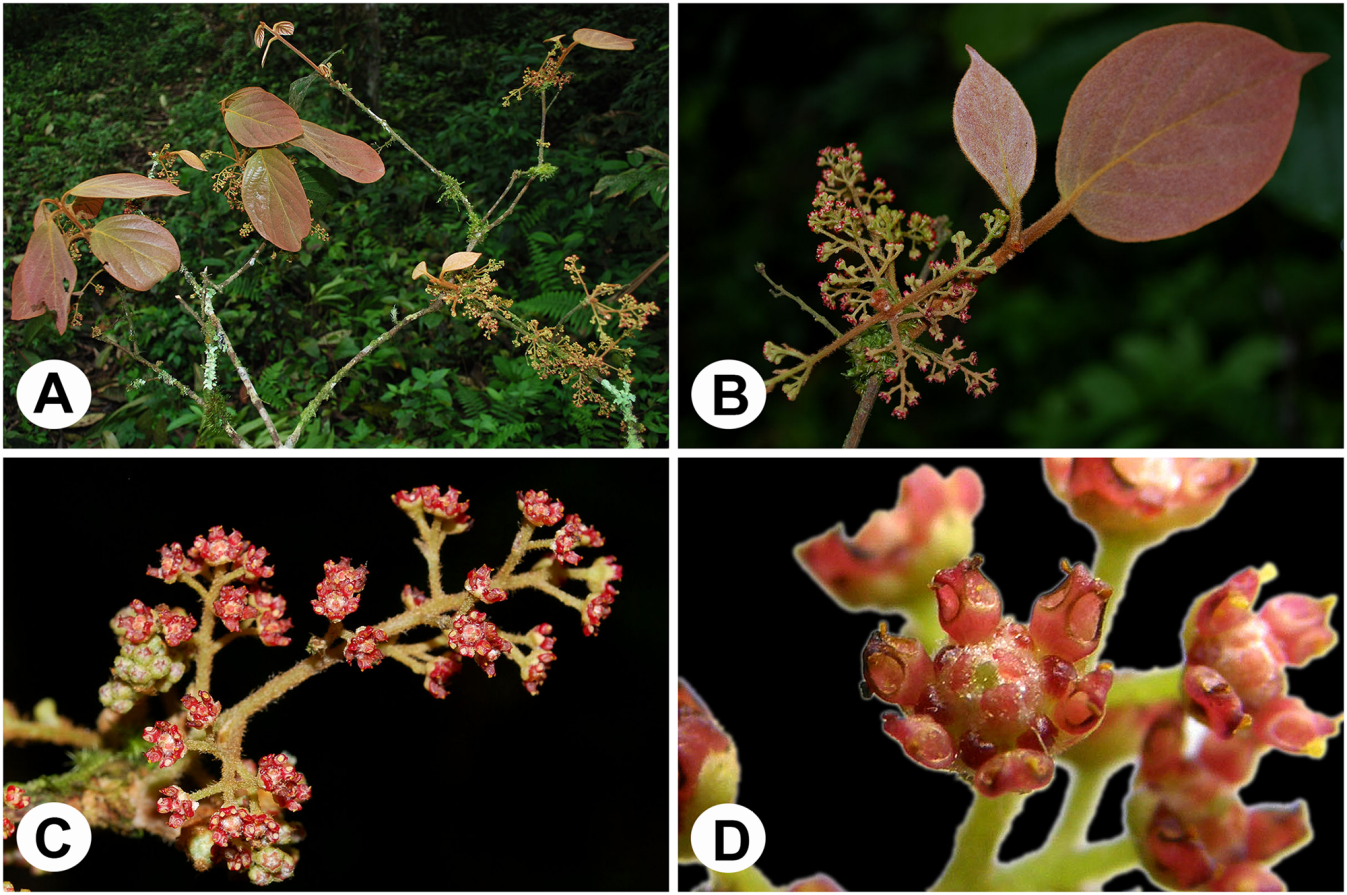
Macromorphology of *Syndiclis marlipoensis*. A, a floral branch; B, tip of a branch with inflorescences; C, lateral view of an inflorescence; D, apical view of a flower.

We collected floral materials of *Syndiclis* for developmental studies for over ten years in southern Yunnan of China. Trees of *Syndiclis* were rarely found in the field. In this study, four species of *Syndiclis* were observed, photographed, and collected, i.e. *Syndiclis marlipoensis* (Fig 2A–2D), *S*. aff. *marlipoensis*, *S*. *fooningensis*, and *S*. *anlungensis*. *Syndiclis* aff. *marlipoensis* is close to *S*. *marlipoensis*, but it probably represents a separate species because it has different floral structures as shown in this study. Materials of *Syndiclis* for anatomic studies were also collected in Guizhou and Guangxi, China. Trees of *Syndiclis* were very rare in the southern China, and we collected floral materials from a few fixed trees. Voucher specimens and pickled materials of flowers were deposited in Herbarium (PE), State Key Laboratory of Systematic and Evolutionary Botany, the Chinese Academy of Sciences, and materials of *Potameia* are deposited in the Missouri Botanical Garden (MO).

Materials of young inflorescences at various developmental stages and mature flowers were collected during 2006 and 2015 (Vouchers see Table 1). Floral materials were fixed in FAA (mixture of formalin, ethanol, and glacial acetic acid) in the field. For purpose of longer preservation, the materials were immersed in new FAA when they were transferred to the laboratory.

**Table 1:** Locality and voucher specimens of plant materials.

Taxon	Locality	Date	Collection
*Syndiclis* aff. *marlipoensis*	Maguan Co., Yunnan Prov., China	Apr. 12^th^, 2014	Liu B., Lin Q. & Jiang L. 2134 (PE)
*Syndiclis* aff. *marlipoensis*	Maguan Co., Yunnan Prov., China	May 15^th^, 2011	Liu B. 1317 (PE)
*Syndiclis marlipoensis*	Malipo Co., Yunnan Prov., China	May 6^th^, 2011	Liu B. 1282 (PE)
*Syndiclis marlipoensis*	Malipo Co., Yunnan Prov., China	Jan. 28^th^, 2015	Guo X.B. s.n. (PE)
*Syndiclis anlungensis*	Anlong Co., Guizhou Prov., China	June 8^th^, 2012	Liu B. 1538 (PE)
*Syndiclis fooningensis*	Napo Co., Guangxi Prov., China	May 23^rd^, 2011	Liu B. 1346 (PE)
*Potameia chartacea*	Toamasina, Madagascar	Nov. 4^th^, 1992	van der Werff H. 12835 (MO)
*Potameia micrantha*	Toamasina, Madagascar	Oct. 27^th^, 1992	van der Werff H. 12777 (MO)
*Potameia microphylla*	Fianarantsoa, Madagascar	Oct. 10^th^, 1992	van der Werff H., Malcomber S.T., Gray B. & Rapanarivo S.H.J.V. 12655 (MO)
*Potameia sp*.	Fianarantsoa, Madagascar	Oct. 11^th^, 1992	van der Werff H., Malcomber S.T., Gray B. & Rapanarivo S.H.J.V. 12682 (MO)

We investigated the floral structures and ontogeny of the genus *Syndiclis* using both light microscopy and scanning electron microscopy. For anatomical studies, floral materials were immersed in ethanol (75%), then observed and dissected to expose the primordia of floral organs under a light microscope (LM). Flowers of different developmental stages and metamorphic variation were selected for further observations. These materials were then dehydrated in an ethanol series, critical point dried, fixed to stubs, and coated with gold palladium. The materials on the stubs were observed and photographed in a HITACHI S-4800 scanning electron microscope (SEM) at 10.0 kV at the State Key Laboratory of Systematic and Evolutionary Botany, Institute of Botany, Chinese Academy of Sciences.

For microtome sections, the dehydrated materials were transferred into xylene and embedded in paraffin. The samples were sectioned with a rotary microtome (4 μm thick). The sections were placed onto microscope slides, and stained with safranin-fast green. Observations were made and micrographs were taken under a Leica DM 5000 B light microscope. Images were edited using Adobe Photoshop CS.

We follow the terminology in Buzgo et al. [16]. For the tribe Cryptocaryeae, a panicle or determinate thyrse is a determinate monopodial main shoot bearing lateral shoots in which each shoot is terminated by a flower and repeats the branching pattern of the bearing shoot. The cross zone is the tissue forming on the adaxial side of a lateral organ (leaf or carpel) that renders the entire structure ascidiate, corresponding to cross meristem.

## Results

### *Syndiclis marlipoensis* [Figs 3A–4C]

**Fig 3:**
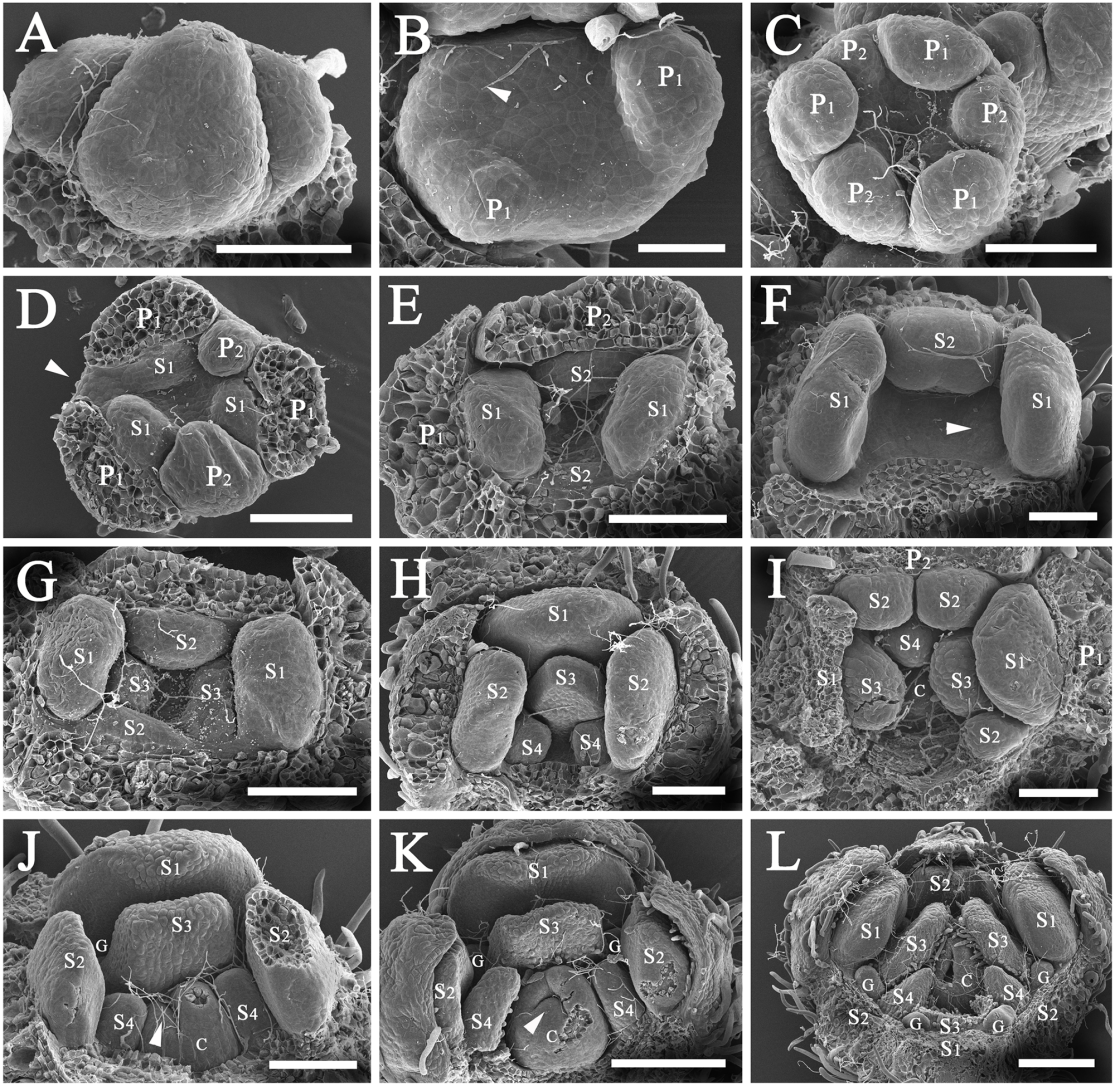
Development of flowers of *Syndiclis marlipoensis*. A, a cyme consisting of three flower buds, the triangular SAMs develop into trimerous flowers while the elliptic SAMs develop into dimerous flowers; B, two of the three primordia of the first tepal whorl, arrow marking the position of a developing tepal (P1); C, three established primordia of the second tepal whorl (P2) showing different development rates of the primordia; D, three established primordia of the first androecial whorl (S1), arrow indicating the position of a tepal (P2) which is expected to occur, leading to a flower possessing five tepals, the two established tepal primordia showing different prominence; E, a dimerous flower bud, the established primordia of the second androecial whorl (S2); F, a dimerous flower bud, stamens of the third androecial whorl not initiated while the first and second whorl of stamens well established, arrow pointing to a minute hump in the position of the third whorl of stamens; G, a dimerous flower bud, the third whorl of stamens initiated; H, a dimerous flower bud, the fourth whorl of stamens initiated; I, a flower bud with irregular merosity, two androecial primordia (S2) initiated in the position of a stamen which is expected to occur; J, a dimerous flower bud, arrow indicating the ovular protuberance; K, a dimerous flower bud, arrow pointing to the ovule not covered by the carpel; L, a trimerous flower, the carpel flanks approximating one another, the ovule enclosed. Scale bars: B: bar = 50 μm; A, C–J: bar = 100 μm; K and L: bar = 200 μm.*Abbreviations*. C, carpel; G, gland; P1, the outer whorl of tepals; P2, the inner whorl of tepals; S1, the first/outermost whorl of stamens; S2, the second whorl of stamens; S3, the third androecial whorl including either fertile stamens or staminodes; S4, the fourth androecial whorl including staminodes.

**Fig 4:**
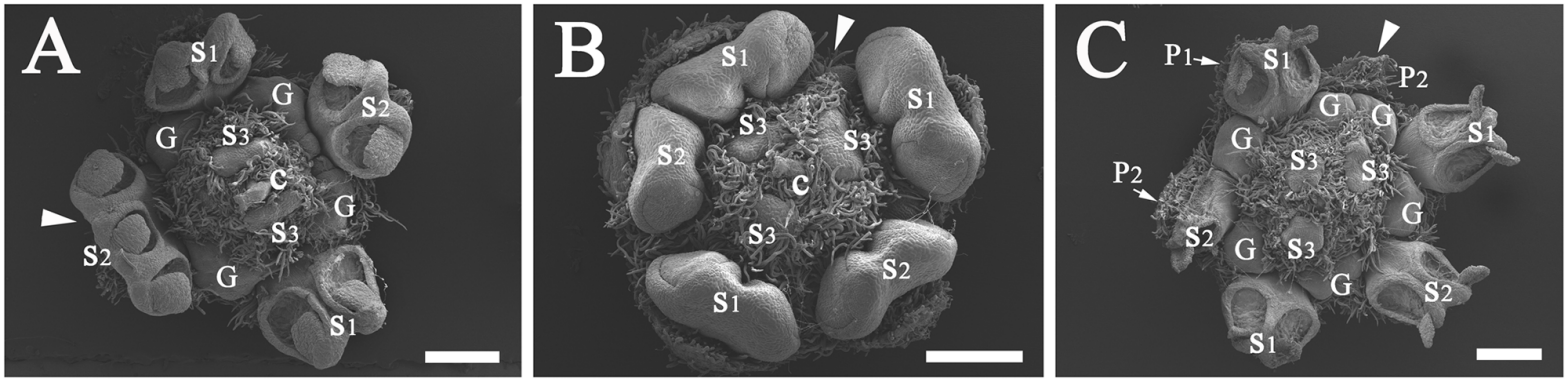
Variation of merosity in *Syndiclis marlipoensis*. A, a dimerous flower, arrow indicating a stamen of the second androecial whorl (S2) showing three locules; B, a complex-whorled flower with trimerous plus dimerous whorls, having five tepals and five fertile stamens, arrow pointing to a missing row of floral organs, leading to the loss of a tepal (P2), a stamen (S2), and a staminode (S4); C, an irregular trimerous flower, arrow indicating a poorly developed tepal of the inner tepal whorl (P2), the adjacent stamen of the second androecial whorl (S2) in the same series missing. Scale bars: A–C: bar = 500 μm. *Abbreviations*. C, carpel; G, gland; P1, the outer whorl of tepals; P2, the inner whorl of tepals; S1, the first/outermost whorl of stamens; S2, the second whorl of stamens; S3, the third androecial whorl including either fertile stamens or staminodes.

This species lives in southeastern Yunnan, China, and flowers twice a year. It initiates its flower buds in late November, and blooms from mid-April to late May. Floral materials collected on January 28^th^ were found to be at various developmental stages, but no open flower was found. The species also blooms from mid-September to mid-October, but it remains unclear when the flower buds are initiated for the autumn.

The small thyrsoid inflorescence consists of several small cymes, each of which comprises two or three flowers. The small cymes are alternate on the inflorescence axis. The terminal flower blooms first, lateral flowers blooming later. The flower bud is rather small and compact, and the floral organs are tightly clustered in the species, which caused difficulties when we dissected the flower buds at early developmental stages.

Flower buds develop asynchronously, so that they are at different developmental stages in inflorescences collected at the same time. The flower develops acropetally. The shoot apical meristems (SAMs) of the floral buds are enlarged at an early stage of initiation, they appear oblong or nearly triangular (Fig 3A and 3B). Protuberances are formed at both ends of the oblong SAM or at the three angles of the triangular SAM, which give rise to the primordia of the first whorl (P1). The primordia of this whorl are initiated asynchronously and show different degrees of prominence (Fig 3B). Then, a protuberance emerges between any two earlier initiated primordia, forming the primordia of the second whorl (P2) (Fig 3C). These three primordia also develop asynchronously (Fig 3C and 3D). Sometimes, a primordium of the second whorl was not initiated (Fig 3D). These primordia produce the two whorls of tepals.

The primordia of the first androecial whorl (S1) are alternate to the second whorl (P2) (Fig 3D). The primordia of the second androecial whorl (S2) parallel to those of the second perianth whorl (P2) are alternate to those of the first androecial whorl (S1) (Fig 3E and 3F). The primordia of the third and fourth whorls (S1 & S2) develop into fertile stamens. The primordia of the fifth and the sixth whorls (S3 & S4) are opposite to those of the third and fourth whorls (S1 & S2), respectively (Fig 3G and 3H), but developmental stasis frequently occurs. The primordia of the fifth and sixth whorl are not initiated when the primordia of the third and fourth whorls are well-developed (Fig 3F), a phenomenon which is frequent in dimerous flowers. The primordia of the fifth and the sixth whorls produce the staminodes.

The carpel primordium is not initiated before the staminodial primordia of the fourth whorl (S4) have become rather prominent (Fig 3H). At the beginning, the carpel is initiated as a protuberance at the centre of the SAMs (Fig 3I). Subsequently, a concavity forms on the slope of the upper to lateral surface, and the cross zone becomes apparent. The ovular primordium forms a protuberance at the cross zone (Fig 3J and 3K). At this time, glands emerge on both sides of each primordium of the fifth whorl (S3), and pubescence appears on stamens and tepals (Fig 3K). Then, the ovule primordium develops and becomes recurved, forming the anatropous ovule. The two flanks of the carpel come close to each other and enclose the ovule (Fig 3L). The staminodes of the third androecial whorl enclose the ovary at anthesis, only leaving the stigma slightly exposed.

Mature flowers possess two whorls of tepals, four androecial whorls including two whorls of stamens and two whorls of staminodes, and a central pistil. The outer two whorls of stamens (S1 & S2) are introrse and fertile, while the inner two whorls (S3 & S4) become staminodes. Anthers are normally two-locular, rarely three-locular (Fig 4A). Although there are usually four to six tepals, and four to six fertile stamens, four or five tepals/stamens are frequently encountered (Fig 4A–4C), but never more than six tepals/stamens. The terminal flower of a cyme usually possesses six tepals and six stamens, but the lateral flowers mostly produce four or five tepals and four or five stamens. Sometimes, two primordia in the place of one primordium develop (Fig 3I). When blooming, the staminodial third and fourth androecial whorls (S3 & S4) enclose the ovary, while the stigma is slightly exserted.

Tepals are sparsely pubescent on the abaxial side, and densely pubescent on the adaxial side. Stamens are pubescent at the proximal portion and nearly glabrous at the distal portion. Staminodes of the third androecial whorl are pubescent only at the proximal portion while glabrous on the distal portion on the abaxial side, but completely pubescent on the adaxial side. Staminodes of the fourth androecial whorl are pubescent. The pistil is glabrous.

### *Syndiclis* aff. *marlipoensis* [Figs 5A–7C]

**Fig 5:**
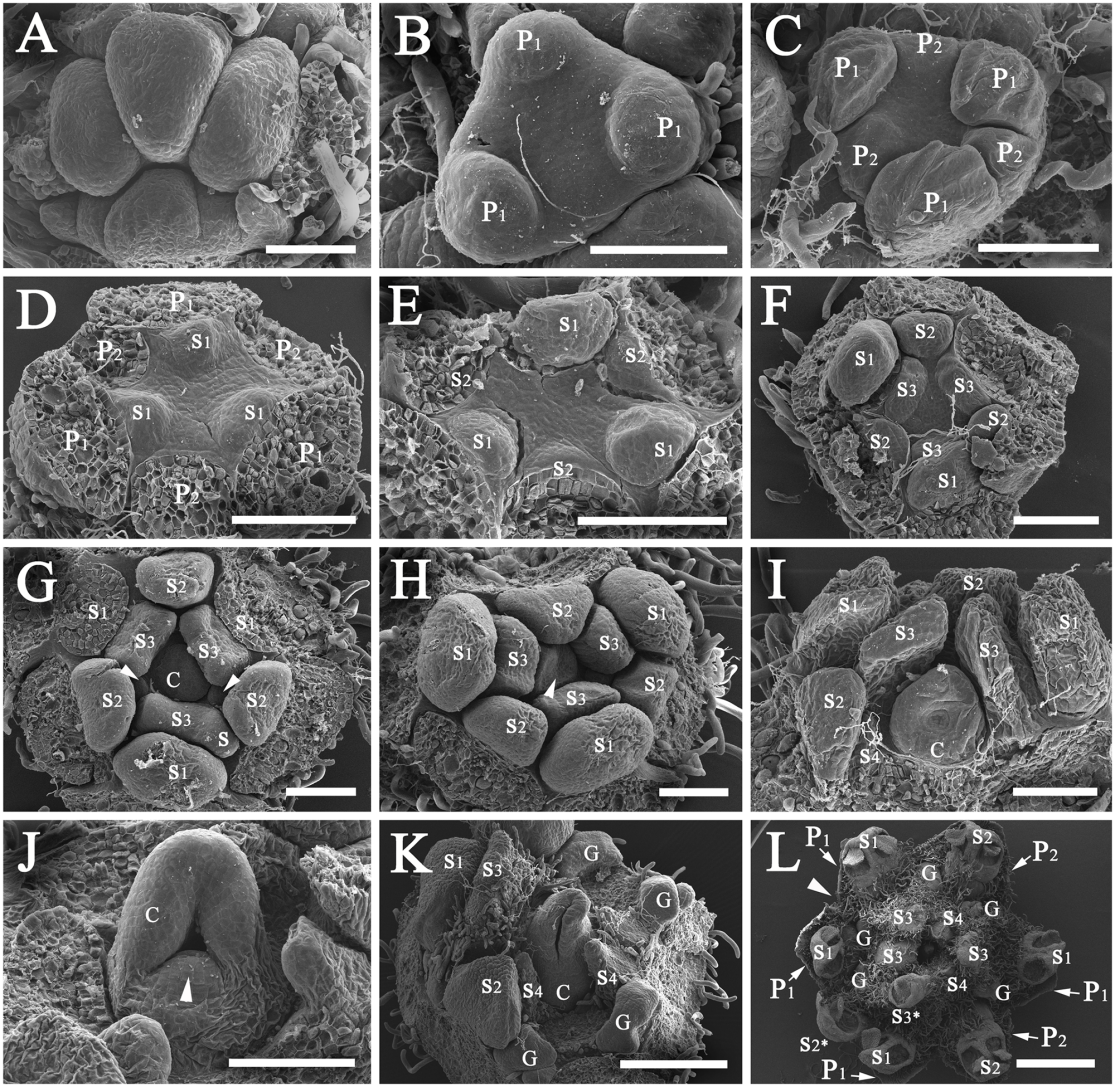
Development of flowers of *Syndiclis* aff. *marlipoensis*. A, a cyme consisting of three flower buds, showing the triangular SAMs; B, three tepal primordia (P1) initiated at different rates; C, three inner tepal primordia (P2) initiated at different rates; D, three primordia of the first androecial whorl (S1) initiated; E, three primordia of the second androecial whorl (S2) initiated; F, three primordia of the third androecial whorl (S3) initiated; G, the carpel primordium initiated, arrow pointing to the staminodial primordium of the fourth androecial whorl (S4), a stamen of the third androecial whorl fused to a supernumerary stamen of the second androecial whorl; H–I, the lateral side of the carpel with concavity; J, the carpel flanks approximating one another, arrow indicating the ovular protuberance; K, fusion of the carpel flanks resulting in the enclosure of the ovule; L, a flower bearing seven tepals and seven fertile stamens, S2* and S3* representing two one-locular stamens, arrow marking the missing orthostichy leading to the loss of a tepal of the inner tepal whorl (P2), a stamen of the second androecial whorl (S2), and a staminode of the fourth androecial whorl (S4). Scale bars: A–J: bar = 100 μm; K: bar = 300 μm; L = 1 mm. *Abbreviations*. C, carpel; G, gland; P1, the outer whorl of tepals; P2, the inner whorl of tepals; S, a supernumerary staminal organ; S1, the first/outermost whorl of stamens; S2, the second whorl of stamens; S3, the third androecial whorl including either fertile stamens or staminodes; S4, the fourth androecial whorl including staminodes.

**Fig 6:**
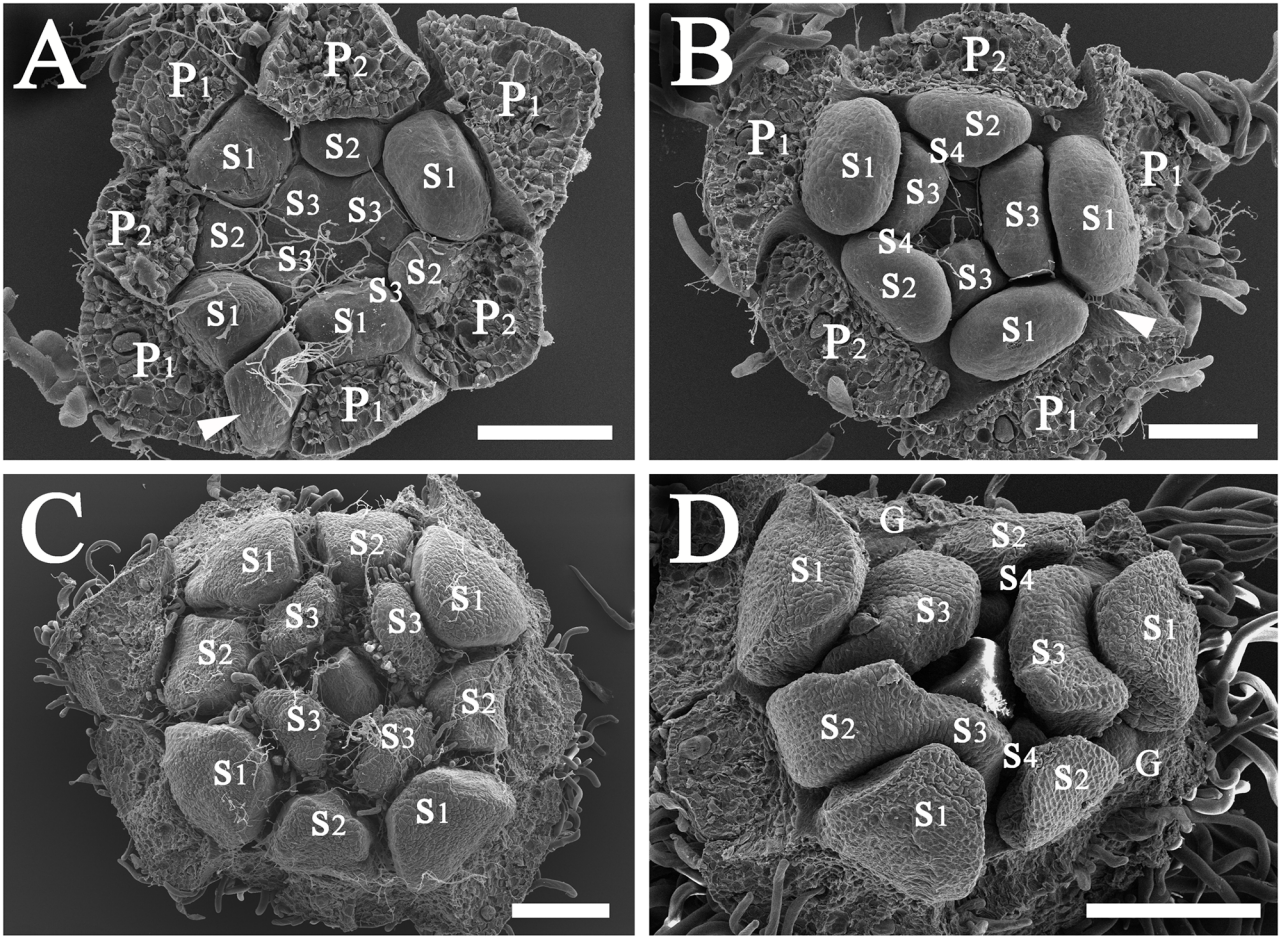
Variation patterns of flowers in *Syndiclis* aff. *marlipoensis*. A, an irregular tetramerous flower bud, arrow highlighting a poorly developed tepal of the inner tepal whorl (P2), the stamen of the second androecial whorl (S2) in the same orthostichy missing; B, a complex-whorled flower with trimerous plus dimerous whorls, arrow indicating a missing orthostichy including a tepal of the inner tepal whorl (P2), a stamen of the second androecial whorl (S2), and a staminode of the fourth androecial whorl (S4); C, a regular tetramerous flower bud; D, a trimerous flower, showing fusion of two adjoining stamens from the third androecial whorl (S3) and the second androecial whorl (S2). Scale bars: A and B: bar = 100 μm; C and D: bar = 200 μm.*Abbreviations*. G, gland; P1, the outer whorl of tepals; P2, the inner whorl of tepals; S1, the first/outermost whorl of stamens; S2, the second whorl of stamens; S3, the third androecial whorl including either fertile stamens or staminodes; S4, the fourth androecial whorl including staminodes.

**Fig 7:**
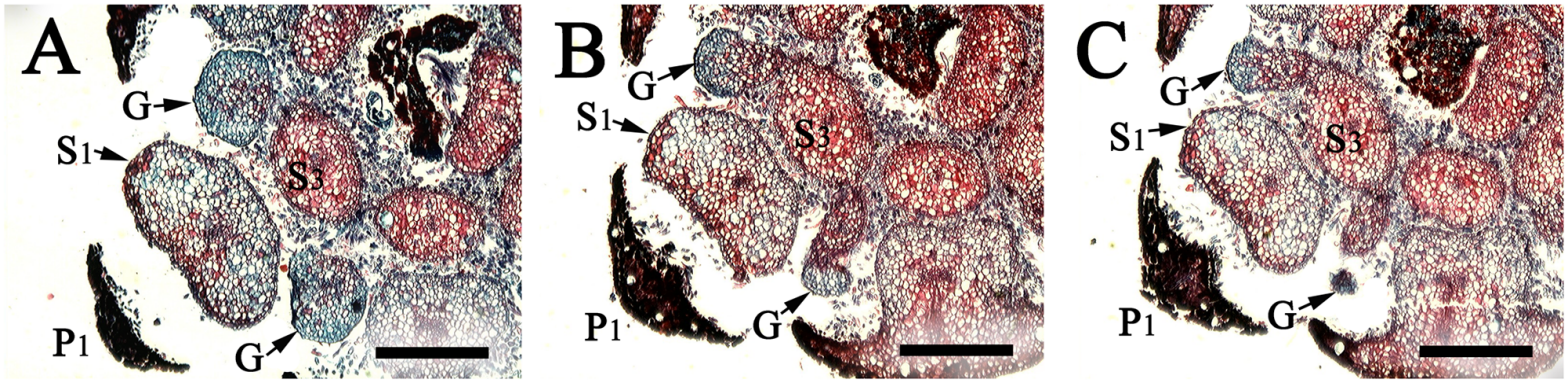
Cross sections of flowers of *Syndiclis* aff. *marlipoensis* showing the two lateral glands (G) attached to the base of filaments of the third androecial whorl (S3) and separate from the first androecial whorl (S1) and the first tepal whorl (P1). Scale bars: A–C: bar = 500 μm.

In southeastern Yunnan, China, this species flowers twice a year. Its flower buds are initiated in mid-December, and flowering occurs from mid-April to late May. Floral materials were found to have various developmental stages on April 12^th^, but there were few open flowers. It is unknown when the flower buds are initiated in the autumn, but it is clear that the species also blooms between mid-September and mid-October.

The thyrsoid inflorescence consists of several subopposite or alternate cymes along the axis, each cyme usually comprises three to five flowers. Flower buds develop asynchronously, so that different developmental stages are found on the same plant at a given time. The flowers develop acropetally as in *S*. *marlipoensis*, i.e. floral organs are initiated from the outer whorl to the inner whorl.

The floral SAMs are enlarged, usually appearing triangular or elliptic (Fig 5A), with three (occasionally four) protuberances, one at each corner, forming the spaced primordia of the first whorl (P1).These primordia develop asynchronously and are unequal in size (Fig 5B and 5C). Subsequently, three primordia of the second whorl (P2) are initiated between every two of the three primordia of the first whorl. Also, the three primordia of the second whorl are different in prominence (Fig 5C). These two whorls of primordia develop into tepals.

Then, the primordia of the third whorl (= first androecial whorl, S1) are initiated (Fig 5D).They are opposite to those of the first whorl (P1) and alternate to the second whorl (P2). The primordia of the fourth whorl (= second androecial whorl, S2) are opposite to the second whorl (P2) and alternate with the third whorl (S1) (Fig 5E). The third and the fourth whorls (S1 & S2) develop into fertile stamens. The fifth and the sixth whorls (S3 & S4) normally develop into staminodes (Fig 5F and 5G).

The carpel primordium is initiated almost at the same time as the primordial initiation of the sixth whorl (S4) and its developmental process is basically the same as that of *S*. *marlipoensis* (Fig 5G–5K). The third whorl of androecial organs (i.e. staminodes S3) appears complanate-linear at anthesis, the apical portion is incurved at maturity and encloses the pistil while leaving the stigma exposed (Fig 5L). The fourth androecial whorl of staminodes (S4) is much smaller than the third whorl of staminodes. Glands, which occur on both sides of the third whorl of stamens (S3), appear irregular-globose at maturity.

There are usually six tepals and six fertile stamens, occasionally seven or eight, rarely five (Figs 5L and 6A–6C). The development of primordia of the outer whorl impacts on the development of the primordia of the inner whorl on the same orthostichy in this species. If one primordium of the second whorl (P2) is poorly developed, then the adjacent primordia of the fourth and sixth whorls (S2 & S4) in a same row are also poorly developed or even lost (Fig 6A). On the contrary, if a primordium of the first or second whorl (P1/P2) is well developed, then its adjacent primordia in the same row are also well developed. The sum of the first and second whorl of stamens (S1 & S2) is equal to the number of tepals (P1 & P2). Rarely, two adjoining stamens of the second and third whorls (S3 & S4) are fused together (Figs 5G and 6D). Anthers are usually two-locular, but occasionally only one-locular (Fig 5L: S2* & S3*).

The flowers are small, and the floral organs are tightly packed. The flower normally possesses two whorls of tepals and four androecial whorls, which develop acropetally. The first and second androecial whorls (S1 & S2) are fertile, the third androecial whorl (S3) sometimes so (Fig 5L), more frequently complanate-linear when sterile, curved inwards distally and enclosing the pistil at maturity. Each of the stamens/staminodes of the third androecial whorl bears two lateral glands at the base (Fig 7A–7C). This species develops a fourth androecial whorl (S4) which is sterile. These staminodes are lanceolate and much smaller than the staminodes of the third whorl (S3). The third and fourth whorls of staminodes closely enclose the pistil and leave the stigma only slightly exposed when blooming.

Tepals are sparsely pubescent on the abaxial side, and densely pubescent on the adaxial side. Stamens of the outer two androecial whorls are only pubescent at the base. Staminal organs of the third androecial whorl, if present, are only pubescent at the base. The staminodes of the third androecial whorl, if present, are pubescent at the proximal portion and glabrous at the distal portion on the abaxial side, and completely pubescent on the adaxial side. The staminodes of the fourth androecial whorl are pubescent. The pistil is glabrous.

### *Syndiclis fooningensis* [Fig 8A–8C]

**Fig 8:**
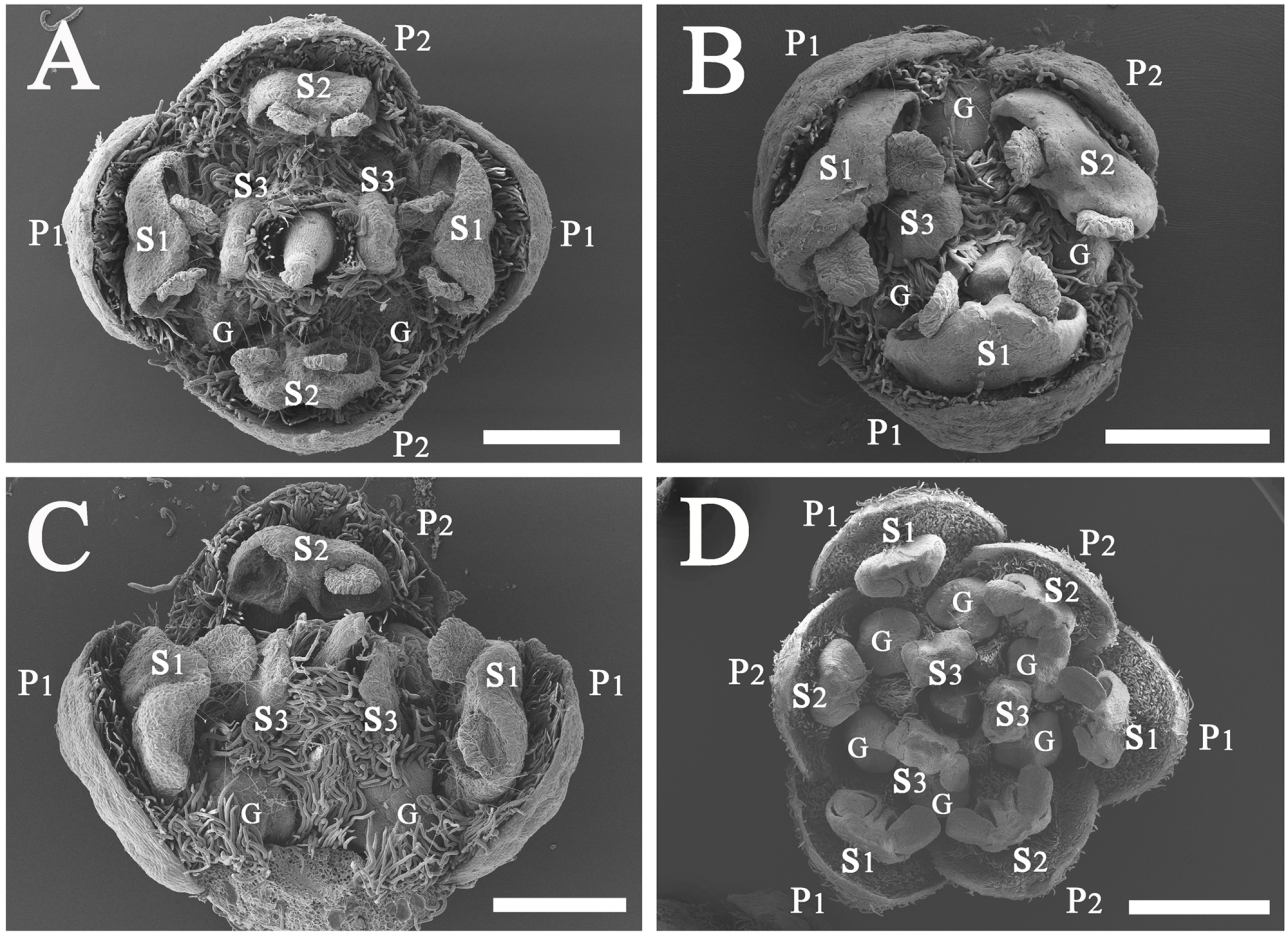
Structural variation of flowers in *Syndiclis fooningensis* (A–C) and *S*. *anlungensis* (D). A, a dimerous flower, bar = 500 um; B, a dimerous flower with one series of floral organs aborted, bar = 500 um; C, lateral view of a dimerous flower with removal of one tepal and its inner stamen displaying enlarged glands and staminodes, bar = 500 um; D, a trimerous flower, bar = 1 mm.

Flowers are small, 1.5–2 mm in diam., rounded or slightly compressed (Fig 8A–8C). The flowers are dimerous (Fig 8A and 8C), but sometimes the dimerous flower loses one orthostichous row. The flower then appears to be a ‘trimerous flower’ which actually consists of a monomerous whorl and a dimerous whorl (Fig 8B). The dimerous flower possesses four tepals in two whorls (Fig 8A and 8C). The tepals are densely pubescent on their inner side. There are four fertile stamens belonging to the first and the second androecial whorl. The filaments are pubescent or only pubescent at the base. The anthers are two-locular, introrse. Stamens of the third androecial whorl are sterile and erect. Two glands are inserted at the basal portion of both sides of the filament. Staminodes of the fourth androecial whorl are minute and lanceolate. The ovary is attenuate to the top forming a style which is elongated between the two erect staminodes; the stigma is small.

Tepals are almost glabrous on the abaxial side, and densely pubescent on the adaxial side. Fertile stamens are only pubescent at the proximal portion. Staminodes of the third androecial whorl are pubescent at the proximal portion and glabrous at the distal portion on the abaxial side, and completely pubescent on the adaxial side. The staminodes of the fourth androecial whorl are pubescent. The pistil is glabrous.

### *Syndiclis anlungensis* [Fig 8D]

Flowers are usually trimerous, slightly bigger than in *S*. *fooningensis*, 2.5–3 mm in diam. (Fig 8D). Each flower has six tepals in two whorls, densely pubescent on both sides. There are nine fertile stamens in three whorls. Anthers are two-locular. The fertile stamens of the first and second androecial whorls are introrse. Stamens of the third androecial whorl are laterorse or slightly extrorse, bearing a gland on each side of the filament. Stamens of the fourth androecial whorl are minute, sterile, and densely pubescent. Stamens are pubescent, the pubescence is denser at the proximal portion than at the distal portion. Staminodes of the fourth whorl are pubescent on the abaxial side. The ovary is glabrous and ovoid, and possesses a style attenuate to the apex.

### *Potameia* 

Mature flowers are very small, usually less than 2 mm in diam. when blooming, circular or elliptic in cross section, and dimerous. Flowers are so simplified that sometimes there are just two stamens belonging to the first androecial whorl (S1) and possessing one-locular anthers. Staminodes of the fourth androecial whorl (S4) and sometimes the third androecial whorl (S3) are absent. The central pistil is conical and relatively large.

#### *Potameia chartacea* [Fig 9A and 9B]

**Fig 9:**
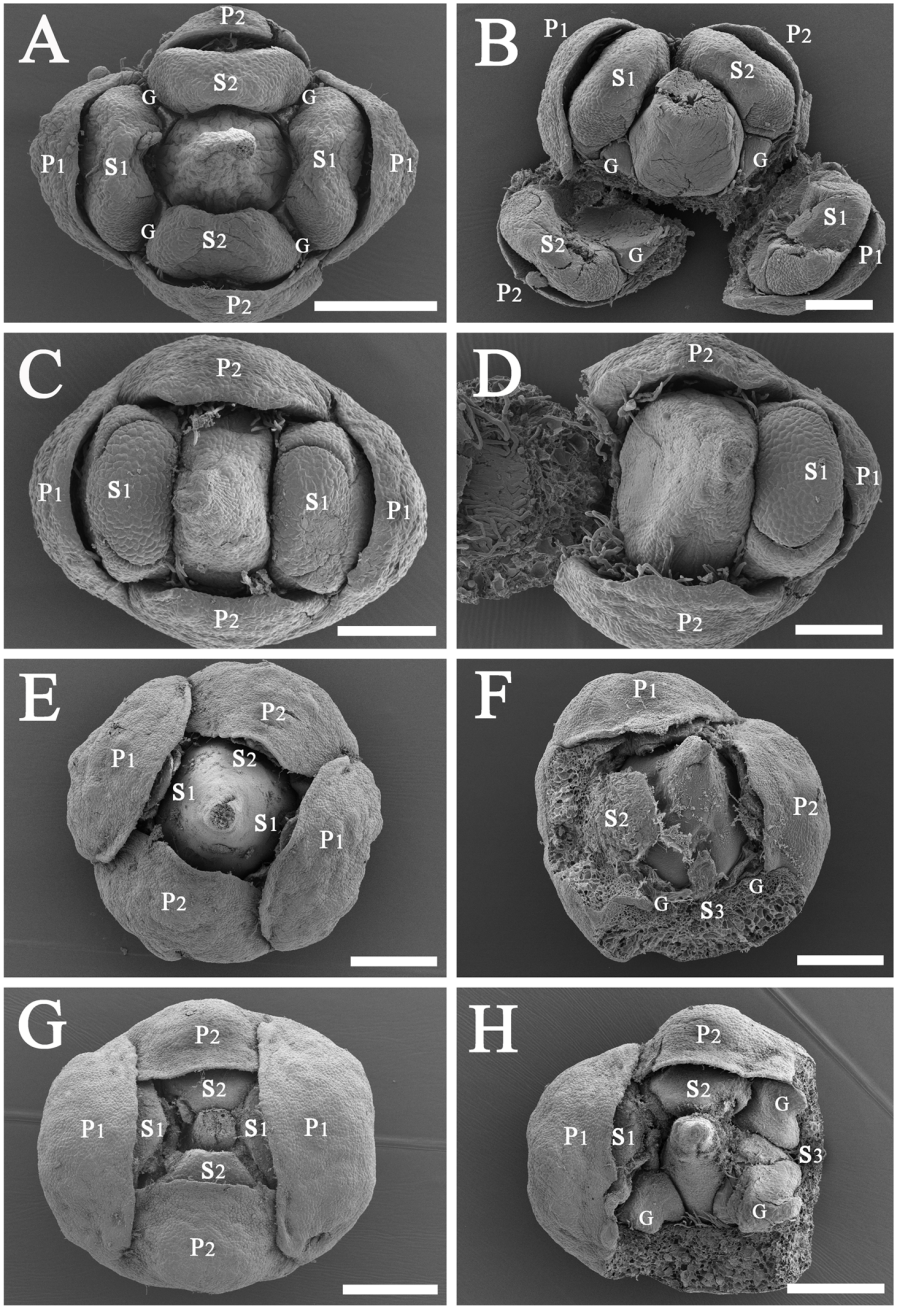
Structural variation of flowers in *Potameia*. A, apical view of a dimerous flower of *P*. *chartacea*, bar = 500 μm; B, lateral view of a dissected flower of *P*. *chartacea* displaying the glands and two-locular anthers, bar = 200 μm; C, apical view of a flower of *P*. *micrantha* displaying the one-locular anthers, bar = 200 μm; D, a dissected dimerous flower of *P*. *micrantha* lacking stamens and glands of the 3^rd^ whorl, bar = 200 μm; E, apical view of *P*. *microphylla* displaying the included stamens and the exposed pistil, bar = 500 μm; F, lateral view of a flower of *P*. *microphylla* displaying the minute stamens and glands of the 3^rd^ whorl, bar = 500 μm; G, apical view of a flower of *P*. sp., bar = 500 μm; H, lateral view of a dissected flower of *P*. *sp*. displaying the stamens and glands of the 3^rd^ whorl, bar = 500 μm.

Mature flowers are elliptic in in cross section, ca. 1.3 mm in diam. They are dimerous, and have four tepals, four stamens (S1 & S2) which are introrse and bear two-locular anthers. The staminodes of the third whorl (S3) and the fourth whorl (S4) are absent. Nevertheless, glands are present in a position between the stamens of the first whorl (S1) and the large pistil. The pistil is conical and attenuate to the apex; the stigma is not enlarged. Floral organs of this species are almost glabrous, only tepals are sparsely pubescent on the adaxial side.

#### *Potameia micrantha* [Fig 9C and 9D]

Mature flowers are elliptic in cross section, ca. 0.9 mm in diam., dimerous, with four tepals, two stamens (S1) which are introrse and have one-locular anthers. The stamens/staminodes of the second whorl (S2), the third whorl (S3) and the fourth whorl (S4) are absent. Glands are absent. The central pistil is conical and large, sometimes compressed. Floral organs of this species are almost glabrous, and tepals are only sparsely pubescent at the proximal portion on the adaxial side.

#### *Potameia microphylla* [Fig 9E and 9F]

Mature flowers are circular in cross section, ca. 1.9 mm in diam., dimerous, and have four tepals and four stamens (S1 & S2) which are introrse and possess two-locular anthers. The staminodes of the third whorl (S3) are minute and those of the fourth whorl (S4) are absent. Glands are inconspicuous. The central pistil is conical and large, and much longer than the stamens. Flowers are almost glabrous, tepals are sparsely pubescent at the base on the adaxial side, stamens and staminodes are sparsely pubescent at the base.

#### *Potameia* sp. [Fig 9G and 9H]

Mature flowers are elliptic in cross section, ca. 1.6 mm in diam., dimerous, and have four tepals and four stamens (S1 & S2) which are introrse and possess two-locular anthers. The staminodes of the third whorl (S3) are minute and those of the fourth whorl (S4) are absent. Glands are round and prominent, positioned at both sides of the staminodes of the third whorl (S3). The pistil is conical, and slightly longer than the stamens. Flowers are glabrous, only tepals and stamens are sparsely pubescent at the base.

A key to the four sampled species of *Potameia* based on floral characters is listed following:

1. Flowers with two stamens; anthers one-locular …………*P*. *microphylla*
1. Flowers with four stamens; anthers two-locular
  2. Staminodes absent …………………………………… *P*. *chartacea*
  2. Staminodes present
    3. Glands inconspicuous; pistils much longer than stamens …………………………………… *P*. *microphylla*
    3. Glands round and prominent; pistils slightly longer than stamens …………………………………… *P*. sp.

## Discussion

### (1) Floral structure of *Syndiclis*

Because of a number of well-known reasons, e.g. few collections in the herbaria and lack of field investigations targeted at these species, variation of a few important taxonomic characters within the genus *Syndiclis* was incompletely known or even erroneously recorded in the literature. Hooker [17] erected the genus *Syndiclis* mainly based on the one-locular anthers, Li & Pai [18] examined the Chinese species, and arrived at the conclusion that anthers of the genus are usually two-locular, and one-locular anthers are rare and caused by fusion of the two locules of the two-locular anthers. Despite this, variation of some other characters remains poorly known, e.g. merosity and organ number of flowers. We observed variation and ontogeny of the four Chinese species of the genus, and try to give a more detailed description of character variation of the genus *Syndiclis* in the following.

Flowers of the genus are dimerous (Figs 3E–3H, 3J, 3K and 4A), or trimerous (Figs 3C, 3L and 5B–5H), or tetramerous (Fig 6C), or have mixed merosity with monomerous and dimerous, or dimerous and trimerous, or trimerous and tetramerous whorls alternating in a flower (Figs 3D, 4B, 5L, 6A and 6B). Mature flowers possess tepals, stamens, staminodes, and a pistil.

#### Tepals

Tepals of the genus *Syndiclis* are in two whorls, equal or subequal, minute, triangular, stiff, and pubescent. Previous studies only recorded flowers possessing four, five or six tepals in the genus [18,19], whereas we also found occasional flowers with three, seven or eight tepals. Variation of tepal number depends on the species; it is variable in some species whereas it is fixed in others. While flowers of *S*. *anlungensis* (at least of the collections examined here) always possess six tepals, it is rather common to see flowers of *S*. *marlipoensis* having four, five or six tepals. Three tepals are sometimes observed in *S*. *fooningensis*, and seven or eight tepals in *S*. *aff*. *marlipoensis*.

#### Stamens

There are four androecial whorls. The outer two whorls of stamens (S1 & S2) are fertile and introrse with two-locular or rarely one-locular anthers. The third whorl (S3) is usually sterile but sometimes fertile in *S*. aff. *marlipoensis*. The organs of the third androecial whorl are linear and complanate if sterile, incurved apically and covering the pistil excepting the stigma for pollination. Each filament of the third whorl of stamens or staminodes (S3) bears a pair of glands at the lateral sides (Fig 7A–7C).

Flowers of *Syndiclis* are rather small and stiff. Therefore, it is rather difficult to dissect them in herbarium specimens and to observe the structural variation of flowers of the genus. It is not always possible to determine exactly the variability of stamen number and which whorl the fertile stamens belong to. As a result, previous studies recorded the fertile stamen number as ranging from four to six but do not clearly state which whorl these fertile stamens belong to. Our observations using SEM clarified this question.

Fertile stamens of the genus range from three to nine. Sometimes all or some stamens of the third androecial whorl are also fertile. Stamen number is variable depending on merosity and on how many stamens of the third androecial whorl are fertile. In *S*. *anlungensis*, stamens of the third whorl are all fertile, and merosity is trimerous, so that there are nine stamens. In *S*. *marlipoensis*, *S*. aff. *marlipoensis*, and *S*. *funingensis*, stamen number is variable because of variation of floral merosity. Variation of stamen fertility in the third androecial whorl increases the number lability in *S*. aff. *marlipoensis*.

Flowers of *Syndiclis* are rather compact, and the fertile stamens have a stout and pubescent filament with the apical portion enlarged toward the anther. Anthers are valvate, usually two-locular, rarely three-locular, and one-locular anthers are also found in *S*. aff. *marlipoensis*. Stamens of the first and second whorl are introrse, and those of the third whorl latrorse or extrorse when fertile.

#### Staminodes

It is well known that a normal flower of the Lauraceae usually has four different kinds of floral organs, i.e. tepals in two whorls, fertile stamens in three whorls, staminodes in one whorl, and a central unicarpellate pistil [2,16]. In *Syndiclis*, staminodes are found not only in the fourth androecial whorl, but also the third androecial whorl may be partially or completely staminodial. Thus the number of staminodes per flower ranges from two to eight, depending on floral merosity and on how many are reduced in the third androecial whorl. The staminodes of the fourth androecial whorl are minute and lanceolate, usually concealed by pubescence, and shorter than the pistil. If the organs of the third androecial whorl are reduced to staminodes, they are oblong and subequal to the pistil or slightly shorter, pubescent on their adaxial side, and glabrous at the upper portion on their abaxial side. The staminodes of the third androecial whorl are incurved and come closer to each other at the top, enclosing the style.

Li & Pai [18] stated that there were four staminodes in the genus, and this character was stable. Our new observations, however, clearly show that there are two types of staminodes in *Syndiclis*, ranging from two to eight in number. In addition, Li & Pai [18] noticed that the arched staminodes cover the ovary. According to our observations, the staminodes of the third androecial whorl are only sometimes incurved to cover the ovary but not always. They belong to the third androecial whorl, not the fourth androecial whorl. The minute staminodes of *Potameia* belong to the third androecial whorl as well. They are much shorter than the style, much smaller than the staminodes of the third whorl in *Syndiclis* but morphologically comparable to the staminodes of the fourth androecial whorl in the latter. In this respect, *Syndiclis* is distinguished from *Potameia*. According to Rohwer et al. [10] and our phylogenetic study of the *Beilschmiedia* group (unpubl. data), *Potameia* is neither related to *Syndiclis* nor to *Sinopora*, but to *Beilschmiedia* species from Madagascar. As a result, it is reasonable to say that the reduction in flower morphology observed in *Syndiclis* and *Potameia* is a case of parallel evolution.

The staminodes are sometimes important in floral biology [20]. Functionally the staminodes of many core Lauraceae are similar to the glands of the fertile stamens of the third whorl in secreting nectar at anthesis. The staminodes function at the female stage and the glands follow during the male stage according to observations in the core Lauraceae [20]. In the *Beilschmiedia* group, these structures tend to be diversified. In most *Beilschmiedia* species, these structures are similar to those in the core Lauraceae. But in *Syndiclis*, the staminodes of the third androecial whorl enclose the ovary at anthesis, and do not appear to secrete nectar but function as a protection of the ovary. We did not observe movements of the staminodes of the third androecial whorl.

#### Glands

In most of the Lauraceae, there are two glands at the base of each filament of the stamens of the third whorl, but variations do occur. In *Brassiodendron*, *Chlorocardium* Rohwer et al., *Phyllostemonodaphne* Kosterm., and *Urbanodendron* Mez, all fertile stamens are found to bear glands at the base of the filaments [2]. In *Anaueria* Kosterm., *Hexapora*, and *Williamodendron* Kubitzki et H.G. Richt. and a few species of *Endiandra*, *Licaria* Aubl., and *Mezilaurus* Kuntze ex Taub., the glands are completely lacking [2]. In some species of *Endiandra* and *Pleurothyrium* Nees, the glands are distinctly enlarged, surrounding the base of all stamens, or even confluent into a glandular cushion [2,21]. Li & Pai [18] stated that the glands were attached to the filaments of the first whorl of stamens in the Chinese *Syndiclis*, but this is certainly not the case. Our observations suggest that the glands still occur at the base of the filaments of the third whorl of stamens.

The glands of the third androecial whorl and normally globose/subglobose. In many samples of *S*. aff. *marlipoensis*, glands occur in different places in the flower, and they are variable in number and size. Such a variation is not common in *S*. *marlipoensis*, which further supports the view that *S*. aff. *marlipoensis* is a different species. Very rarely the glands appear irregularly inserted, but never regularly inserted at the base of the filament of the first androecial whorl.

#### Pistil

The pistil of *Syndiclis* normally consists of one carpel. Its ventral suture is clearly visible when young but indiscernible when mature. The pistil is conical and attenuate into a short style, the stigma is slightly exserted above the other floral organs. The ovary is glabrous. In the species examined here, the style of *Syndiclis* is relatively shorter than that in *Potameia* relative to the ovary. In a few cases, the pistil consists of two carpels that are opposite at the ventral side and free or fused at the basal portion. Whether this bicarpellate status bears evolutionary implications and how this morphological change takes place requires further studies.

### (2) Lability of floral organs in *Syndiclis*

The number of floral organs is labile in *Syndiclis*. The floral whorls are relatively stable in a given species, but primordia usually vary in number, leading to changes in the number of floral organs. The shape of the SAMs appears to be related to the number of floral organs. In most cases, the SAM primordia are non-equilateral triangles. The three primordia are initiated at the three corners, and then the alternate primordia of the inner whorls develop. The common trimerous flowers are developed by this way (Figs 3A, 3C, 3L and 5A–5H). However, sometimes the primordia are arranged in a circle or square when initiated. In this case, the outer whorl consists of four primordia, with the inner whorls alternatingly developing four primordia, such that a tetramerous flower forms (Fig 6C). Sometimes, a flower bud appears spindly, with only two primordia in the outer whorl (at opposite ends of the spindle). As each of the inner whorls alternatingly produces two primordia, a dimerous flower develops (Figs 3E–3H, 3J, 3K and 4A).

Occasionally, a complex-whorled pattern is found. The smaller primordia of the outer whorl are sometimes not initiated, and one floral organ is lacking from the whorl (Fig 3D), which leads to a difference in the number of floral organs between two adjacent whorls. If the outer whorl of tepals initiates three primordia, and the adjacent inner whorl develops only two primordia, then the flower appears five-tepaled with mixed base number, i.e. three (trimerous) plus two (dimerous) tepals (Figs 3D, 4B and 6B). If the outer whorl is initiated with four primordia, and the adjacent inner whorl develops three primordia, then the flower appears seven-tepaled with a mixed base number, i.e. four (tetramerous) plus three (trimerous) tepals (Fig 5L).

The stamen primordium of the second androecial whorl (S2) is usually well developed when the parallel primordium of the second perianth whorl (P2) is well developed. Otherwise, the stamen primordium of the fourth whorl (S2) disappears when the primordium of the second perianth whorl (P2) in the same row does not develop (Figs 4B, 5L, 6A and 6B). This leads to an alternating pattern of adjacent whorls with different base numbers, with alternative numbers of floral organs from the outer to the inner whorls for the two tepal whorls and four stamen whorls, i.e. three, two, three, two, three, two, the flower appears “pentamerous” (actually trimerous plus dimerous) with five tepals, five stamens, and five staminodes (Figs 4B and 6B).

Irregular variability in floral organ number, not affecting entire orthostichies, may occur as well. Sometimes, the number of floral organs is increased by two primordia replacing the one primordium which normally occurs (Fig 3I). This situation occurred irregularly, and did not result in an increase in the number of floral organs in the adjacent inner whorl or every other inner whorl.

In the *Beilschmiedia* group, there are regular trimerous flowers, e.g. *B*. *yunnanensis* Hu and *Sinopora hongkongensis* (N.H. Xia et al.) J. Li et al., dimerous flowers in a few species of *Syndiclis* [18,19], and flexible merosity in *B*. *appendiculata* [22]. Staedler & Endress [23] discovered four types of floral phyllotaxis in the Laurales, i.e. Fibonacci spiral, simple-whorled, complex whorled, and irregular. The simple whorled floral phyllotaxis refers to those flowers with uniformly isomerous whorls, and is rare in pluricarpellate families but pervasive in unicarpellate families such as the Lauraceae [23]. The simple whorled phyllotaxis is common in the core Lauraceae including the *Persea* Mill. group as Buzgo et al. [16] reported, and in *Cryptocarya* and most *Beilschmiedia* species. The complex-whorled phyllotaxis is defined as lacking isomerous floral whorls, but merosity increases or decreases between whorls, as a result of the replacement of one primordium by two or more collateral primordia, so-called double or multiple positions [23]. This situation also occurs in the Chinese *Syndiclis* (Fig 6I). Irregular phyllotaxis is rare in *S*. *marlipoensis*.

Flowers are relatively “open”, and floral phyllotaxis (organ arrangement) and merism (organ number) are plastic in primitive angiosperms [24,25]. It is true that organ number and arrangement of flowers are the main sources of variation in the Lauraceae. Merosity of flowers is extremely variable in the *Beilschmiedia* group. In this study, we found dimerous, trimerous, tetramerous, even mixed-merous including trimerous plus dimerous and tetramerous plus trimerous flowers in *Syndiclis* (Figs 6C, 6D, 6H, 6L, 7A–7C, 8B, 8C, 8G, 8H, 8L and 9A–9D). In *Caryodaphnopsis* Airy Shaw, the flowers are usually trimerous, but sometimes tetramerous flowers are found (pers. observ.). *Chlorocardium* also bears tetramerous flowers in addition to irregular flowers and occasional tri- or "pentamerous" flowers [26]. Even in the core Lauraceae it is not uncommon to find some tetramerous flowers in inflorescences otherwise bearing normal trimerous flowers. Examples from several taxa are available on http://lauraceae.myspecies.info/gallery/tetramerous.

### (3) Implications of floral ontogeny of *Syndiclis*

Ontogenetic observations showed additional variation of flower morphology in *Syndiclis*. The two species of *Syndiclis* investigated here have flowers where floral organs are initiated asynchronously. A tepal primordium initiated early in the inner whorl is much more prominent than the other primordia of the same whorl, but is as conspicuous as the retarded tepal primordium in the outer whorl (Figs 3B–3D, 5B and 5C). This developmental pattern was also observed in the core Lauraceae, e.g. *Cinnamomum* Schaeff. [27], *Umbellularia* (Nees) Nutt. [28], and *Persea americana* Mill. [16]. In all cases, the floral organs are not initiated simultaneously but both plastochrons and divergence angles are unequal between whorls. The flowers of the Lauraceae are defined as whorled because the plastochrons and divergence angles are equal or nearly so within a whorl but markedly different between two adjacent whorls [29].

It seems that floral merosity is programmed in the SAMs at a very early stage of the floral buds before the initiation of floral organs. In many cases, the flowers are dimerous when the SAMs are oblong or ellipsoid (Fig 3E and 3F), but trimerous when the SAMs are triangular (Fig 5A–5C). Any changes in the early phases may result in parallel changes of merosity in the flowers. Given that there are six sets of orthostichous floral organs at the initiation of the shoot apical meristem (SAM) of the flower, the flower appears “pentamerous” (trimerous plus dimerous) if one set is lost (Figs 4B and 6B). The flower appears “heptamerous” (tetramerous plus trimerous), or tetramerous if the SAM contains eight sets of orthostichous floral organs (Figs 5L and 6C). If two adjoining sets of primordia are lost in the SAM, then the trimerous flower appears dimerous (Fig 3E–3H, 3J and 3K). Sometimes, the outermost tepal primordium of an abortive orthostichy is not completely missing but remains relictual (Figs 4C and 6A).

### (4) A comparison of floral characters between *Syndiclis* and other genera in the *Beilschmiedia* group

The genus *Syndiclis* displays a wide range of variation of floral morphology compared to other genera of the *Beilschmiedia* group, and contains almost all variation patterns in all other genera (Table 2). In *Syndiclis*, the flowers are rather compact; they possess minute tepals, and sessile or nearly sessile stamens and glands. This kind of miniature flowers is also seen in other small genera, e.g. *Endiandra*, *Hexapora*, *Potameia*, *Sinopora*, and *Yasunia*, as well as in some (particularly some African) species of *Beilschmiedia*. Most other species of *Beilschmiedia*, in contrast, have typical trimerous flowers as those of *Cryptocarya*, *Persea*, *Machilus*, *Phoebe*, and *Cinnamomum*. The miniature flowers of *Syndiclis* show extremely high variability in merosity, including not only dimerous and trimerous, but also tetramerous and mixed merous arrangements, whereas the flowers are either trimerous or dimerous in other genera. The staminodes seem unusual in *Syndiclis*; those of the third whorl are complanate-linear and enclose the ovary, and those of the fourth whorl resemble to those of the third whorl in *Potameia*. Staminodes enclosing the ovary are also found in *Aspidostemon*, *Hexapora* and *Sinopora*. Flowers of both *Syndiclis anlungensis* and *Sinopora hongkongensis* are trimerous, but they differ by the presence of glands (present in *Syn*. *anlunensis* vs. wanting in *Sin*. *hongkongensis*) and number of fertile stamens (nine in *Syn*. *anlunensis* vs. six in *Sin*. *hongkongensis*). The South American genus *Yasunia* includes a species with trimerous flowers and one with dimerous flowers, but in contrast to *Sydiclis* the staminodes are columnar in *Yasunia*. Though variation and reduction of floral organs are generally observed in the *Beilschmiedia* group, it is unusual to find that the glands are conspicuous in *Syndiclis* (Table 2). Probably, the enlarged glands are to guide pollinators during pollination. To better understand the mechanism, further studies are necessary for these species with miniature flowers.

**Table 2:** A comparison between *Syndiclis* and its relatives.

Character	*Beischmiedia*	*Endiandra*	*Potameia*	*Sinopora*	*Syndiclis*	*Yasunia*	*Brassiodendron*	*Triadodaphne*	*Hexapora*
Merosity	trimerous, rarely dimerous	trimerous, rarely dimerous	dimerous	trimerous	dimerous, trimerous, tetramerous, mixed merous	dimerous or trimerous	trimerous, sometimes irregular	dimerous or trimerous	trimerous
Tepal number	6, rarely 4	6, rarely 4	4	6	3−8	6 or 4	6	4 or 6	6
Stamen number	9 (6 or 8)	3 (6)	4 (2)	6	3−9	3 or 4, rarely 2	6, 5 or 4	3	6
Stamen whorls	I, II, III (I, II)	III (II, III)	I, II	I, II	I, II (III)	I (II)	I, II	III	I, II
Staminodia number	3 or 4	absent or 3	2	6	2−8	3 or 2	absent	3	6
Staminodia shape	stipitiform, often with sagittate apex	stipitiform	stipitiform, minute	thick	staminodes including two types, the 3^rd^ whorl complanate-linear, and the 4^th^ whorl minute and lanceolate	columnar	-	minute, stipitiform, tepaloid or thickly conical	thick
Anther locule number	2	2 or 1	2, rarely 1	2	2, rarely 1 or 3	2	2	2	2
Anther locule direction	the 1^st^ and 2^nd^ whorls introrse, the 3^rd^ whorl extrorse	extrorse	apical- introrse	apical	the 1^st^ and 2^nd^ whorls introrse, the 3^rd^ whorl laterorse or extrorse if fertile	introrse-apical or lateral-distal	(extrorse) latrorse(introrse)	latrorse or extrorse	extrorse
Glands enlarged	no	sometimes	no	absent	yes	no	no; minute or absent	no; mostly absent	absent
Veinlet reticulations	fine, coarse	fine	coarse	fine	fine	coarse	fine	coarse	fine
Distribution	pantropical	tropical Asia, Oceania	Madagascar	China	Bhutan, China	Ecuador, Peru	Moluccas, New Guinea, Australia	Borneo, New Guinea, Solomon Islands	Malay Peninsula
Reference	[12]	[12]	[12]	[12]	this study	[12]	[2]	[30]	[2]

### (5) Evolution of floral characters in the tribe Cryptocaryeae

The diversity of flowers in the *Beilschmiedia* group merely indicates a secondary diversification if the phylogeny of the Lauraceae is considered. Reduction in organ number and size in the flower may have resulted from the selective pressure in shaping a miniature flower in adaptation to some new pollination syndromes.

The Lauraceae are unusual in the evolution of a few important characters, e.g. ovary position and organ number. The ovary is inferior or at least enclosed in a deep receptacle and the fruit is enclosed in a receptacular cupule in the basal Lauraceae (e.g. *Hypodaphnis* Stapf) and in outgroups, e.g. Hernandiaceae and Monimiaceae [2,31,32]. These features have been more or less retained in the *Cryptocarya* group [10], e.g. in *Dahlgrenodendron*, *Aspidostemon*, *Eusideroxylon*, *Potoxylon*, and *Cryptocarya*. The superior ovary in the *Beilschmiedia* group and the core Lauraceae is thus secondary. This has evolved twice in the *Beilschmiedia* clade and in the non-cryptocaryoid Lauraceae if the genus *Cassytha* L. is considered to be a reversal.

It is worth pointing out that the evolution from an inferior to a superior of ovary in the Lauraceae is contrary to the general evolutionary trend in angiosperms according to traditional botanical doctrine [33]. On the other hand, floral organ number seems to evolve from more or less fixed in the *Cryptocarya* group to rather labile in the *Beilschmiedia* group, while it is well known that the evolution of flowers in angiosperms proceeds from indefinite to fixed organ number [24]. The flowers of the basal lineages of the tribe Cryptocaryeae evolved more or less fixed floral organ number probably because they gained special genetic control by selective pressure (e.g. pollinators). The lability of floral organ number in the *Beilschmiedia* group is probably caused by a breakdown of the genetic control or loss of the special selective pressure. To better understand this mechanism, it is necessary to conduct detailed investigations on the pollination system of *Syndiclis* and other members of the *Beilschmiedia* group.

Evolutionary changes of floral organ number in the *Beilschmiedia* group seem irregular according to the most recent phylogenetic study [10]. Reduction of fertile stamens has taken place independently in different lineages. For instance, *Yasunia* possesses reduced flowers with three or four fertile stamens. The genus is nested within a clade including a few *Beilschmiedia* species from South America having flowers with nine fertile stamens [10], suggesting that the reduced flower of *Yasunia* was derived from the nine-stamened flower. *Potameia* from Madagascar bears reduced flowers with four fertile stamens. The genus is nested within a clade consisting of a few African *Beilschmiedia* species having usually nine stamens (sometimes six stamens in *B*. *madagascariensis* (Baill.) Kosterm.). This implies that the reduced flower of *Potameia* is a simplified form of the African *Beilschmiedia*. Both *Yasunia* and *Potameia* have dimerous flowers, but the two genera belong to two different clades [10], demonstrating that dimerous flowers independently evolved from trimerous flowers at least twice in the *Beilschmiedia* group. Moreover, *Sinopora* has flowers possessing six stamens. It appears to be sister to a clade including a few Central American *Beilschmiedia* species with nine stamens according to the result of a combined Bayesian *trn*K and ITS analysis [10]. Thus *Sinopora* appears to be another case of loss of fertility in the stamens of the third androecial whorl, a phenomenon well known from some genera in the Core Lauraceae, such as *Aiouea* [34] or *Persea* [35].

## References

[1] Kostermans AJGH. Lauraceae. Reinwardtia. 1957; 4: 193–256.

[2] Rohwer JG. Lauraceae In: Kubitzki K, Rohwer JG, Bittrich V, editors. The families and genera of vascular plants, vol. 2 Berlin: Springer-Verlag; 1993 pp. 366–391.

[3] van der Werff H, Richter HG. Toward an improved classification of Lauraceae. Ann Mo Bot Gard. 1996; 83: 409–418.

[4] Li J, Christophel DC, Conran JG, Li HW. Phylogenetic relationships within the ‘core’ Laureae (Litsea complex, Lauraceae) inferred from sequences of the chloroplast gene *mat*K and nuclear ribosomal DNA ITS regions. Plant Syst Evol. 2004; 246: 19–34.

[5] Trofimov D, Rudolph B, Rohwer JG. Phylogenetic study of the genus *Nectandra* (Lauraceae), and reinstatement of Damburneya. Taxon. 2016; 65: 980–996.

[6] Rohwer JG, Li J, Rudolph B, Schmidt SA, van der Werff H, Li HW. Is *Persea* (Lauraceae) monophyletic? Evidence from nuclear ribosomal ITS sequences. Taxon. 2009; 58: 1153–1167.

[7] Li L, Li J, Rohwer JG, van der Werff H, Wang ZH, Li HW. Molecular phylogenetic analysis of the *Persea* group (Lauraceae) and its biogeographic implications on the evolution of tropical and subtropical amphi-Pacific disjunctions. Amer J Bot. 2011; 98: 1520–1536. 21860056 10.3732/ajb.1100006

[8] Chanderbali AS, van der Werff H, Renner SS. The relationships and historical biogeography of Lauraceae: evidence from the chloroplast and nuclear genomes. Ann Mo Bot Gard. 2001; 88:104–134.

[9] Rohwer J, Rudolph B. Jumping genera: the phylogenetic positions of *Cassytha*, *Hypodaphnis*, and *Neocinnamomum* (Lauraceae) based on different analyses of trnK intron sequences. Ann Mo Bot Gard. 2005; 92: 153–178.

[10] Rohwer JG, de Moraes PLR, Rudolph B, van der Werff H. A phylogenetic analysis of the *Cryptocarya* group (Lauraceae), and relationships of *Dahlgrenodendron*, *Sinopora*, *Triadodaphne*, and *Yasunia*. Phytotaxa. 2014; 158: 111–132.

[11] Van der Werff H. A synopsis of the genus *Beilschmiedia* (Lauraceae) in Madagascar. Adansonia Sér. 3. 2003; 25: 77–92.

[12] Van der Werff H, Nishida S. *Yasunia* (Lauraceae), a new genus with two species from Ecuador and Peru. Novon. 2010; 20: 493–502.

[13] Yang Y, Zhang LY, Liu B, van der Werff H. Leaf cuticular anatomy and taxonomy of *Syndiclis* (Lauraceae) and its allies. Syst Bot. 2012; 37: 861–878.

[14] Liu B. Systematics and biogeography of the subtribe Beilschmiediinae (Lauraceae) in China. Ph.D thesis. Institute of Botany, CAS. 2013.

[15] Liu B, Yang Y, Xie L, Zeng G, Ma KP. *Beilschmiedia turbinata*: a newly recognized but dying species of Lauraceae from tropical Asia based on morphological and molecular data. PLoS ONE. 2013; 8(6): e67636 doi: 10.1371/journal.pone.0067636 23840756 10.1371/journal.pone.0067636 PMC3695919

[16] Buzgo M, Chanderbali AS, Kim S, Zheng ZG, Oppenheimer DG, Soltis PS, et al Floral developmental morphology of *Persea americana* (Avocado, Lauraceae): the oddities of male organ identity. Int J Plant Sci. 2007; 168: 261–284.

[17] Hooker JD. The Flora of British India, vol. 5. L. Reeve and Co.; 1886.

[18] Li HW, Pai PY. *Syndiclis* In: Li HW, editor. Flora Reipublicae Popularis Sinicae, vol. 31, Lauraceae and Hernandiaceae. Beijing: Science Press; 1982 pp. 152–160.

[19] Li J, Xia NH, Li XW. *Sinopora*, a new genus of Lauraceae from south China. Novon. 2008; 18: 199–201.

[20] Rohwer JG. The timing of nectar secretion in staminal and staminodial glands in Lauraceae. Plant Biol. 2009; 11: 490–492. doi: 10.1111/j.1438-8677.2008.00184.x 19470120 10.1111/j.1438-8677.2008.00184.x

[21] Hyland BPM. A revision of Lauraceae in Australia (excluding *Cassytha*). Aust Syst Bot. 1989; 2: 135–367.

[22] Lee SK, Wei YT. *Beilschmiedia* Nees In: Li HW, editor. Flora Reipublicae Popularis Sinicae, vol. 31, Lauraceae and Hernandiaceae. Beijing: Science Press; 1982 pp. 123–149.

[23] Staedler YM, Endress PK. Diversity and lability of floral phyllotaxis in the pluricarpellate families of core Laurales (Gomortegaceae, Atherospermataceae, Siparunaceae, Monimiaceae). Int J Plant Sci. 2009; 170: 522–550.

[24] Endress PK. Origins of flower morphology. J Exp Zool. 2001; 291: 105–115. doi: 10.1002/jez.1063 11479912 10.1002/jez.1063

[25] Endress PK. Structure and relationships of basal relictual angiosperms. Aust Syst Bot. 2004; 17: 343–366.

[26] Rohwer JG, Richter HG, van der Werff H. Two new genera of Neotropical Lauraceae, and critical remarks on the generic delimitation. Ann Mo Bot Gard. 1991; 78: 388–400.

[27] Singh V, Singh A. Floral organogenesis in *Cinnamomum camphora*. Phytomorphology. 1985; 35: 61–67.

[28] Kaspaligil B. Morphological and ontogenetic studies of *Umbellularia californica* Nutl. and *Laurus nobilis* L. Univ Calif Publ Bot. 1951; 25: 115–240.

[29] Endress PK, Doyle JA. Floral phyllotaxis in basal angiosperms: development and evolution. Curr Opin Plant Biol. 2007; 10: 52–57. doi: 10.1016/j.pbi.2006.11.007 17140838 10.1016/j.pbi.2006.11.007

[30] Kostermans AJGH. *Triadodaphne*, a new lauraceous genus from Borneo. Reinwardtia. 1974; 9: 119–121.

[31] Kubitzki K. Hernandiaceae In: Kubitzki K, Rohwer JG, Bittrich V, editors. The families and genera of vascular plants, vol. 2 Berlin: Springer-Verlag; 1993 pp. 334–338.

[32] Philipson WR. Monimiaceae In: Kubitzki K., Rohwer JG, Bittrich V, editors. The families and genera of vascular plants, vol. 2 Berlin: Springer-Verlag; 1993 pp. 426–437.

[33] Singh G. Plant Systematics: An Integrated Approach. New Hampshire: Science Publishers Inc; 2004.

[34] Kubitzki K, Renner S. Flora Neotropica Monographs, vol. 31, Lauraceae I (*Aniba* and *Aiouea*). New York: The New York Botanical Garden; 1982 Pp. 1–124.

[35] Kopp LE. A taxonomic revision of the genus Persea in the Western Hemisphere (Perseae-Lauraceae). Mem New York Bot Gard. 1966; 14: 1–120.

